# Examining the mechanisms underlying the acquisition of animal tool behaviour

**DOI:** 10.1098/rsbl.2020.0122

**Published:** 2020-06-03

**Authors:** Elisa Bandini, Alba Motes-Rodrigo, Matthew P. Steele, Christian Rutz, Claudio Tennie

**Affiliations:** 1 Department for Early Prehistory and Quaternary Ecology, The University of Tübingen, Tübingen 72070, Germany; 2 Centre for Biological Diversity, School of Biology, University of St Andrews, St Andrews KY16 9TH, UK

**Keywords:** animal tool behaviour, learning mechanism, baseline experiment, social learning, tool use, tool manufacture

## Abstract

Despite major advances in the study of animal tool behaviour, researchers continue to debate how exactly certain behaviours are acquired. While specific mechanisms, such as genetic predispositions or action copying, are sometimes suspected to play a major role in behavioural acquisition, controlled experiments are required to provide conclusive evidence. In this opinion piece, we refer to classic ethological methodologies to emphasize the need for studying the relative contributions of different factors to the emergence of specific tool behaviours. We describe a methodology, consisting of a carefully staged series of baseline and social-learning conditions, that enables us to tease apart the roles of different mechanisms in the development of behavioural repertoires. Experiments employing our proposed methodology will not only advance our understanding of animal learning and culture, but as a result, will also help inform hypotheses about human cognitive, cultural and technological evolution. More generally, our conceptual framework is suitable for guiding the detailed investigation of other seemingly complex animal behaviours.

## 1. Introduction

Although the field of ethology continues to mature, how animals' behavioural repertoires are formed and maintained remains under debate. While some argue that in certain cases single mechanisms are at play––such as action copying––this view is inconsistent with a growing body of experimental evidence demonstrating the involvement of multiple drivers. Indeed, as early as 1968, Tinbergen [1] cautioned that a strict dichotomy of innate versus learned (nature versus nurture) behaviour should be avoided, and that researchers should instead embrace the ontogenetic complexity of animal behaviour.

Tool behaviours, including both tool use and tool making, are often regarded as being especially complex in terms of their underlying cognitive and motor processes [2]. The advent of tool behaviour in human history has been argued to be one of the main catalysts of our species' remarkable evolutionary journey [3], motivating broad comparative studies of non-human tool behaviour, social learning and cognitive capacities (e.g. [4]). Thus, an improved understanding of how tool behaviours develop in non-human species, and how they are passed across generations, has implications for a range of fields, including evolutionary anthropology, archaeology, and cultural and technological evolution.

As with most behaviours, it seems unlikely that a single factor is responsible for the emergence and maintenance of most types of tool behaviour. Instead, behaviours likely arise through a combination of genetic predispositions, individual learning and social influences, mediated by environmental context ([1]; see also [5]). Here, we argue that studies investigating the mechanisms giving rise to animal tool behaviours should focus on examining the relative contributions––and ideally the timing––of each of these factors. This issue should be addressed with recourse to experimental approaches rooted in classic ethology (e.g. [6–9]). We believe that robust baselines and social learning experiments must be carried out before conclusions can be drawn about the role of particular mechanisms in the emergence of specific behaviours.

The step-wise methodology presented here builds on pioneering work by the founders of ethology, especially Nikolaas Tinbergen [8,10] and Konrad Lorenz [6,11]. These authors stressed the importance of studying the ontogenetic development of individual behaviours while carefully controlling for subjects' previous experiences, for example, by testing naive hatchlings [7,10]. In more recent years, however, the field seems to have moved away from this powerful approach, despite continued interest in identifying the principal factors contributing to behavioural acquisition.

Of all the factors that may contribute to the emergence of animal tool behaviours, action copying (e.g. imitation) is often singled out as a major––and sometimes the only––driver, especially when discussing the tool repertoires of our closest living relatives, non-human great apes (e.g. [12]). This interest in copying may stem from the fact that much of modern human culture relies on copying mechanisms––that is, the cultural transmission of ‘know-how’ (e.g. [13–16]). Indeed, many modern human tool behaviours have advanced to such a degree that their know-how has become culture dependent and can only be learnt via copying [17]. However, without the use of adequate experimental tests, it is impossible to pinpoint whether copying is also responsible for the acquisition of specific tool behaviours in non-human animals. Below, we describe the most robust methodology, in our view, for pursuing this goal.

## 2. Baseline tests

The experimental conditions we outline in this essay aim to determine if a target tool behaviour is acquired through individual processes (i.e. genetic predispositions and/or trial-and-error learning), social learning (catalysed via the behaviour or behavioural products of others), or indeed specific copying social-learning mechanisms (i.e. action copying). During initial baseline tests, target-naive subjects are given all the materials and opportunities required for the expression of the target tool behaviour, in the absence of social information about the behavioural actions or products. That said, these baseline tests do not occur in an informational vacuum. The experimental provision of materials (e.g. of potential tools near a food task, unless these materials are already available to the subjects without provision) may attract the subjects' attention towards specific objects or locations [18]. Thus, these baselines do not test for the rate of discovery in the absence of enhancement. Instead, they recreate circumstances that social animals will routinely encounter in the wild, where social cohesion, observable food choices and even the artefactual remains left behind by others (e.g. abandoned tools, or debris resulting from tool manufacture and use) likewise enhance some materials over others (so-called ‘cultural founder effects’; [15]).

If naive subjects exhibit the target behaviour in baseline tests, this demonstrates that the know-how of the behaviour is not contingent on copying. Previous studies employing this approach revealed that tool behaviours can indeed spontaneously emerge without direct social input in a range of bird and primate taxa (table 1). This can be owing to genetic predispositions or individual learning (facilitated by environmental context), or most likely an interaction between the two (such behaviours have been previously described as re-innovations [24]).

**Table 1.** Selected examples primate and bird studies (the taxa we work on) employing baseline tests in which at least one naive, captive subject spontaneously expressed a target tool behaviour. The degree of subjects' naivety varies between studies (see column four), which should be taken into consideration when interpreting the results of these studies.

species	reference	results summary	naivety status of subjects
woodpecker finch (*Cactospiza pallida*)	Tebbich *et al*. [19]	Juvenile woodpecker finches developed tool use regardless of whether or not they had seen a tool-using model.	Wild birds were brought into captivity 12 days after hatching, i.e. before they had an opportunity to observe adults using tools (owing to their nest structure, hatchlings cannot see outside the nest).
New Caledonian crow (*Corvus moneduloides*)	Kenward *et al*. [20]	Juvenile New Caledonian crows spontaneously manufactured and used tools regardless of whether they had seen a human demonstrator or not.	Subjects were hand-raised in an aviary. Juveniles developed tool manufacture and use without ever having any contact with conspecific adults or observing any demonstration by humans.
Hawaiian crow (*Corvus hawaiiensis*)	Rutz *et al*. [21]	Juvenile Hawaiian crows held in two social groups started using sticks and other objects as probing tools.	Captive-bred crows were raised in captivity without ever seeing an adult conspecific or human using tools to extract hidden food (as confirmed by keepers); but note that birds could observe the actions of other naive subjects in their social groups.
chimpanzee (*Pan troglodytes*)	Kitahara-Frisch & Norikoshi [22]	A chimpanzee spontaneously started using leafy branches to retrieve juice from an apparatus (sponging).	Captive-born chimpanzees never saw a model performing sponging (no further information available).
gorilla (*Gorilla gorilla gorilla*)	Boysen *et al*. [23]	Gorillas spontaneously used stick tools to fish peanut butter out of an artificial dome, a behaviour that does not resemble any behaviours observed in the wild.	The naivety status of the gorillas was not specified, but note that gorillas have very rarely been observed using tools in the wild.
chimpanzee (*Pan troglodytes*)	Bandini & Tennie [24]	Naive chimpanzees spontaneously started using sticks to scoop floating bread from a container of water.	Captive-born chimpanzees were mother-reared and had no previous experience of retrieving floating food (as confirmed by keepers).

## 3. Updating the baseline methodology

In order to examine the sources of specific animal behaviours, it is necessary to control for subjects' pre-testing experience. Tinbergen and Lorenz tried to control for these factors by testing newly hatched birds (e.g. [7,10]), an approach still in use today (e.g. see [21,25,26]; although note that learning of some behaviours can occur in unhatched bird embryos; e.g. [27]). In the case of viviparous animals, running baseline tests is inherently more challenging, especially as we strongly discourage––for ethical reasons––rearing individuals of social species in isolation. That said, for some captive animals, detailed records on their rearing histories exist and information on prior experiences can often be provided by keepers (e.g. [21,24,28–30]). To avoid false positives during later testing, any behaviour described by keepers should be assumed to be present in the subjects’ repertoire and therefore these subjects should not be considered target naive. To further promote this approach, research facilities should, where possible, keep detailed records on their animals' prior experiences and behavioural repertoires (including access to enrichment materials and opportunities to observe conspecific and heterospecific models, as well as participation in earlier experiments). Furthermore, staff should be briefed to refrain from demonstrating behaviours of interest and from providing target artefacts to potential test subjects.

Determining past experiences of wild animals is much more difficult as subjects usually cannot be followed continuously, and their environment cannot be controlled. Despite these complications, some studies with access to long-term data succeeded in tracking tool innovations, as well as social-learning opportunities for behaviours not previously shown by the groups concerned (e.g. [31,32]).

If the subjects’ naivety has been confirmed, studies should ideally include both baseline and social-learning conditions. Baseline tests should be long enough to allow animals to familiarize themselves with the materials provided, account for changes in motivation levels and provide sufficient time for trial-and-error learning [33]. We suggest that, as a rule of thumb, baseline tests should be at least double the length of any follow-up social-learning conditions. This acknowledges the hypothesized difference in efficiency between individual and social learning (reviewed by [34]), with animals often expressing behaviours faster when they have access to social learning.

Some studies on captive primates have made commendable efforts to include baselines in their experimental designs (e.g. [35–38]). We noticed, however, that in many of these studies, baselines were either carried out with fewer subjects (e.g. *N* = 5 in baseline versus *N* = 11 and *N* = 12 in social conditions; [35]) or baseline subjects were given less time to individually explore solutions, compared to subjects exposed to social-learning opportunities (e.g. 2 h in asocial condition versus 10 h in social conditions, [38]; one asocial trial versus 15 social trials, [37]; for further discussion, see also [39]).

If a target behaviour is found in a baseline test, this does not mean that the behaviour must necessarily lose its status as a ‘cultural’ trait. Even behaviours that emerge during baseline tests can still be considered cultural if at least the frequency of their expression across subjects is influenced by some variant of social learning [40]*.* For example, animals might be socially attracted towards specific components of tool behaviours (e.g. which foods are edible [know-what], or where raw materials to use as tools or for tool manufacture are to be found [know-where]) via local and/or stimulus enhancement. This makes it more likely that they will acquire the target behaviour, resulting in an overall increase in the behaviour's frequency. Following this minimal criterion of culture [40], various animal tool behaviours can be considered cultural (e.g. [24,29,41–43]).

Likewise, it is possible that genetic predispositions channel the emergence of a basic behavioural capacity, while social learning is required for the acquisition of specific additional skills. This could be the case in New Caledonian crows, for example, where captive-bred naive juveniles develop basic stick tool use without opportunities to observe models, but do not exhibit some of the complex tool manufacture behaviours seen in nature, which may require social input [20,44,45].

## 4. Moving beyond baselines

**Figure 1.**
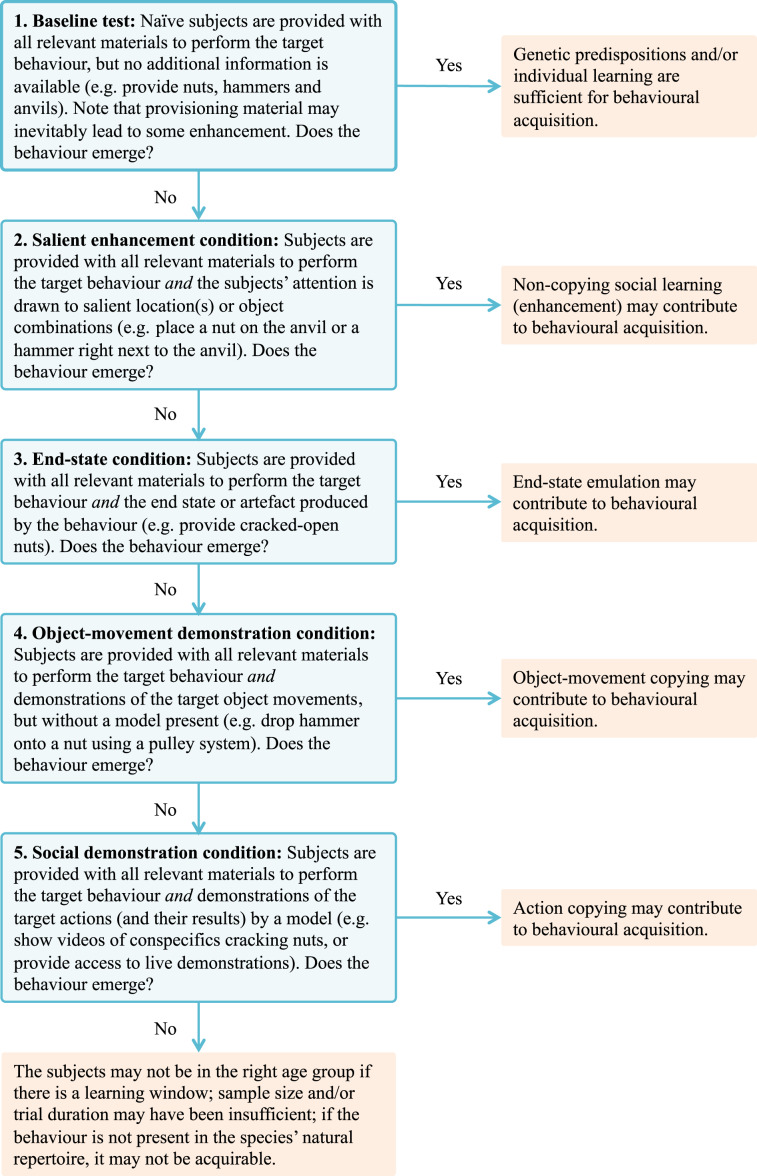
Decision tree with possible outcomes of baseline and social-learning conditions for experimental studies on the sources of animal tool behaviours. For illustration purposes, details of a hypothetical nut-cracking study are included for each condition.

If a behaviour does *not* emerge in an initial baseline test, it may be that some variant of social learning other than enhancement is required for its acquisition. In these cases, baselines should be supplemented with a step-wise series of social-learning conditions (figure 1). The incremental addition of social information will then help determine if social learning is required (e.g. variants of emulation or action copying; [28]).

These additional tests could be carried out using either a within-subject design (i.e. the same individuals are successively tested across all conditions) or a between-subject design (i.e. different individuals are tested in different conditions). Within-subject designs have the advantage of controlling for important confounds––such as individual differences in responsiveness or ability, resulting from factors such as rearing background, previous experience, or age––but require repeated testing of subjects, which may not always be logistically feasible. By contrast, between-subject designs involve shorter (cumulative) testing times for individual animals, but require a larger pool of test subjects, which may raise ethical concerns (although most experiments of this kind are expected to constitute welcome enrichment for captive subjects in research facilities). These trade-offs need to be carefully evaluated on a case-by-case basis, informed by detailed knowledge of the candidate subjects and testing circumstances.

If the target behaviour still does not appear in any of the social-learning conditions, even once a full demonstration has been provided, alternative explanations must be explored. For example, the subjects may already be outside their sensitive learning periods for acquiring the behaviour via individual or socially mediated learning (compare [6]). Another possibility is that, in within-subject designs, subjects' motivation levels did not remain sufficiently high to persevere with the task. In cases in which motivation levels drop, we recommend interspersing test trials with motivation trials, in which a different, easy-to-solve task is presented, but without providing information on the target task solution. Motivation trials need to be designed carefully, however, as they may unintentionally provide social information (e.g. local enhancement). Experiments can also investigate whether particular actions or variants require additional input (e.g. basic tool use may emerge during baseline tests, but idiosyncratic ways of making or holding tools may depend on seeing models). Finally, the species might not be capable of the behaviour, even after full demonstrations are provided [15].

## 5. Conclusion

Although not all behavioural contexts can be meaningfully recreated during experiments in captivity, there is a growing body of evidence demonstrating that, across different species, various tool behaviours can emerge spontaneously in baseline tests. This leaves open the question about which animal tool behaviours may be culture dependent [17]. For example, the apparent complexity of stepped pandanus tool making in New Caledonian crows [45,46] and nut-cracking in chimpanzees [47] makes these behaviours candidate culture-dependent behaviours, although this remains to be explicitly tested. We suggest that researchers interested in examining the sources of animal tool behaviours should also work systematically through the tool repertoires of their target species (e.g. see repertoires of various great ape species described in [48–50]) and test each behaviour separately, following the approach described here (see also [28]). By applying our step-wise methodology, we can systematically investigate the relative contributions of different mechanisms to the development and maintenance of animal tool behaviours, and indeed many other seemingly complex behaviours. This return to one of the key methodologies of classic ethology holds the potential to generate valuable advances across a wide range of disciplines concerned with the behavioural capacities of human and other animals.

## Data accessibility

This article has no additional data.
